# Socioeconomic inequalities in paediatric metabolic syndrome: mediation by parental health literacy

**DOI:** 10.1093/eurpub/ckad028

**Published:** 2023-02-27

**Authors:** Alexander Lepe, Marlou L A de Kroon, Sijmen A Reijneveld, Andrea F de Winter

**Affiliations:** Department of Health Sciences, University Medical Center Groningen, University of Groningen, Groningen, The Netherlands; Department of Health Sciences, University Medical Center Groningen, University of Groningen, Groningen, The Netherlands; Department of Public Health and Primary Care, Centre Environment & Health, KU Leuven, Leuven, Belgium; Department of Health Sciences, University Medical Center Groningen, University of Groningen, Groningen, The Netherlands; Department of Health Sciences, University Medical Center Groningen, University of Groningen, Groningen, The Netherlands

## Abstract

**Background:**

Parental health literacy may explain the relationship between parental socioeconomic status (SES) and paediatric metabolic syndrome (MetS). For this reason, we assessed to what extent parental health literacy mediates the relationships between parental SES and paediatric MetS.

**Methods:**

We used data from the prospective multigenerational Dutch Lifelines Cohort Study. Our sample consisted of 6683 children with an average follow-up of 36.2 months (SD 9.3) and a mean baseline age of 12.8 years (SD 2.6). We used natural effects models to assess the natural direct, natural indirect and total effects of parental SES on MetS.

**Results:**

On average, an additional 4 years of parental education, e.g. university instead of secondary school, would lead to continuous MetS (cMetS) scores that were 0.499 (95% confidence interval (CI): 0.364–0.635) units lower, which is a small effect (*d*: 0.18). If parental income and occupational level were 1 SD higher, on average cMetS scores were 0.136 (95% CI: 0.052–0.219) and 0.196 (95% CI: 0.108–0.284) units lower, respectively; these are both small effects (*d*: 0.05 and 0.07, respectively). Parental health literacy partially mediated these pathways; it accounted for 6.7% (education), 11.8% (income) and 8.3% (occupation) of the total effect of parental SES on paediatric MetS.

**Conclusions:**

Socioeconomic differences in paediatric MetS are relatively small, the largest being by parental education. Improving parental health literacy may reduce these inequalities. Further research is needed into the mediating role of parental health literacy on other socioeconomic health inequalities in children.

## Introduction

Socioeconomic inequalities exist in paediatric metabolic syndrome (MetS),^1–3^ and parental health literacy may partially explain these inequalities. MetS is a cluster of cardiometabolic risk factors (i.e. waist circumference, mean arterial pressure, insulin resistance, triglycerides, and high-density lipoproteins),^4^ which can result in increased cardiometabolic risk later in life.^5^^,^^6^ Due to the long-term impact of MetS, prevention early in life would likely provide favourable consequences over the life course. In order to prevent paediatric MetS, we need a better understanding of the mechanisms linking socioeconomic status (SES) to MetS, such as the role of parental health literacy.

Parental health literacy may be an important modifiable mechanism linking SES to paediatric MetS, but this pathway remains understudied in children. Health literacy can be defined as ‘the degree to which individuals have the capacity to obtain, process, and understand basic health information and services needed to make appropriate health decisions’.^7^ In adults, there is evidence that health literacy mediates the relationship between own SES and health, and that the strength of these relationships vary depending on which proxy of SES is used^8^; education has the strongest relationship with health literacy. Additionally, there is a well-known relationship between parental health literacy and various aspects of children’s health.^9^ Parents with low health literacy are more likely to experience difficulties managing their child’s chronic diseases, and are more likely to incorrectly use medications.^9^ Additionally, parents with low health literacy are more likely to have less optimal health behaviours, e.g. smoking, alcohol, nutrition and physical activity.^9^ These factors contribute to the association between low parental health literacy and children’s obesity, which is a major contributor to MetS,^10^ in children.^9^ Given these relationships, it seems that parental health literacy may mediate the relationship between SES and paediatric MetS.

Evidence on the mediating role of parental health literacy is relevant because it is a modifiable target for preventive interventions to reduce socioeconomic health inequalities in paediatric MetS. Therefore, we aimed to assess the extent to which parental health literacy mediates the relationships between different measures of SES and MetS.

## Methods

### Setting and population

Data were used from the Lifelines Cohort Study, a multi-disciplinary prospective population-based cohort study examining in a unique three-generation design the health and health-related behaviours of 167 729 persons living in the North of the Netherlands.^11^^,^^12^ It employs a broad range of investigative procedures in assessing the biomedical, socio-demographic, behavioural, physical and psychological factors which contribute to the health and disease of the general population, with a special focus on multi-morbidity and complex genetics. A detailed description of the recruitment strategy and data collection can be found elsewhere.^11^ Briefly, Dutch-speaking individuals aged 25–49 years were asked to participate by their physicians. Those who accepted were subsequently asked to invite their family members. Individuals could also self-register through the Lifelines website. The first measurement wave took place between 2007 and 2014 and 2010 and 2014 in adults and children, respectively. During the first (T1) and fourth (T4) measurement wave, participants were asked to fill out questionnaires and, if aged 8 years or older, they also underwent physical exams.

For the purpose of this paper, Lifelines provided the data of 15 016 children aged 0–17 years at baseline whose data could be linked to at least one parent. For this study, we included all 6683 children aged 8–17 years during T4, and they had a mean follow-up duration of 36.2 (SD: 9.3) months. Children under the age of 8 years at T4 (*n* = 2167) were excluded because the components of MetS were not assessed in those children, and children who turned 18 years old during the follow-up (*n* = 1332) were excluded as this study focuses on paediatric MetS. In addition to the 6683 children included in our analysis, 3086 eligible children were lost to follow-up. An additional 1748 children were lost to follow-up but would have likely been excluded due to being too old or too young during T4, because they were aged 0–4 or 16–17 years during T1. Written informed consent was obtained for each participant prior to participating in the cohort. The Lifelines Cohort study is conducted according to the conventions set forth in the Declaration of Helsinki, and it has received approval from the Medical Ethics Committee of the University Medical Center Groningen (METc approval number: 2007/152).

### Procedures

Participants completed questionnaires, physical exams and venous blood draws during both the first and fourth assessment. During the third (T3) assessment, participants only completed questionnaires. Questionnaire data were self-reported and covered various topics including demographics. Physical exams and venous blood draws were conducted by trained research nurses using a standardized protocol.^11^

### Measures

MetS was measured by a continuous MetS (cMetS) score, which was defined using measures of waist circumference, mean arterial pressure, fasting glucose, triglycerides and high-density lipoproteins from T4. Following an approach similar to Eisenmann et al.,^4^ each component of MetS was regressed on age and sex, and their residuals were standardized. Then, a cMetS score was built by summing the standardized residuals of all components. The standardized high-density lipoprotein residuals were reversed due to their inverse relationship with health. The cMetS score indicates how an individual’s cardiometabolic health compares to the rest of our sample.^4^

Parental health literacy was measured at T3 using self-reported answers to the three validated questions from Chew et al.^13^

1. How often do you have trouble understanding your medical situation because you have difficulty with the written information?
2. How sure are you of yourself when you fill out medical forms?
3. How often does someone help you with reading information materials from the hospital or another healthcare provider?

The parents of our participants indicate how often or to what extent these items apply to them on a scale ranging from ‘never/not at all’ (1) to ‘always/very’ (5). We reversed the scores of the first and third questions and then added up the scores of all questions, resulting in a health literacy scale ranging from 3 to 15. This score was then categorized into low (3–12) and adequate (13–15) health literacy, as was done in previous studies using data from Lifelines^14^^,^^15^ and leads to percentages of low and adequate health literacy comparable to those from large-scale health literacy surveys in the Netherlands.^16^ Because Dutch mothers usually spend more time parenting,^17^ we constructed the parental health literacy variable using the mother’s health literacy. If this was missing, the father’s score was used. Additionally, we created an average measure of parental health literacy. If data from both parents were available, we took the mean of their health literacy scores and then dichotomized this into low and adequate health literacy. If data were only available for one parent, that parent’s health literacy was used.

SES was measured using three separate indicators: parental education, occupation and income. These data were obtained from both parents during T1. Education was assessed by asking parents about the highest educational level they attained, with eight potential responses ranging from ‘no education’ to ‘university’. In an approach similar to De Graaf et al.,^18^ these categories were recoded into years of education using the number of years it would take to complete each category by the fastest route possible; e.g. no education, primary school, secondary school and university were coded as 5, 6, 12 and 16 years, respectively. Occupation was coded using the International Standard Classification of Occupations 2008.^19^ This was recoded into Treiman’s Standard International Occupational Prestige Scale, which is a continuous measure of occupational prestige.^20^^,^^21^ For both education and occupation, the highest level from either parent was used. If only one parent was registered in Lifelines, then only data from that parent were used. To construct the measure of equivalized household income (income), net household income was divided by the square root of the number of people living on this income.^22^ Similar to how health literacy was constructed, the mother’s response was used, but the father’s response was used if this was missing.

Parental age was defined as the mean parental age from T1. If we only had records for one parent, then that parent’s age was used. This approach was used because both parental SES and health literacy come from a mixture of maternal and paternal data.

### Statistical analysis

First, we described the characteristics of our sample. We compared the characteristics of the eligible children who participated in T4 and the eligible children that were lost to follow-up. For this comparison, we only included children from the lost to follow-up group who most likely would have been eligible to participate in our study (baseline age 5–15 years). Before conducting any further analyses, we first imputed missing values for our variables. As all missing variables were continuous, we used the predictive mean matching (20 imputations) method from mice (v3.13.0)^23^; we used as predictors of the missing values low-density lipoprotein, total cholesterol, glycated haemoglobin, weight, height, hip circumference, body mass index, systolic blood pressure, diastolic blood pressure, age, sex and parental age.

**Figure 1:**
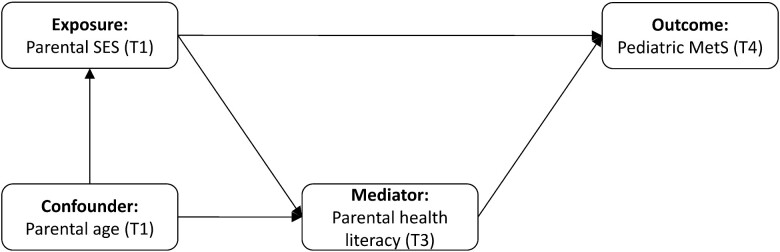
Model demonstrating the direct and indirect pathways connecting parental SES to paediatric MetS

To assess the extent to which parental health literacy mediates the relationships between different measures of SES and MetS (figure 1), we conducted a causal mediation analysis which uses the potential outcomes framework.^24^ This approach defines the causal effect of an exposure as the contrast of outcomes that would be observed under different exposure and mediator values.^24^ To achieve this contrast of potential outcomes, we expanded our dataset using an imputation procedure, from the medflex (v0.6-7) package for R, which results in the same individuals being evaluated at different levels of our exposure.^25^ After expanding the dataset, we fit natural effects models using the medflex package.^25^ Both the imputation procedure used to expand the dataset and the natural effect models were adjusted for baseline parental age. We also conducted sensitivity analyses in which we used average parental health literacy instead of the mostly maternal measure of parental health literacy. All data preparation and analyses were conducted using R version 4.0.2.^26^

## Results

### Sample characteristics

One-fifth of the children included in the sample came from households with low parental health literacy (table 1). When parental health literacy was defined as the average of both parents’ health literacy scores, the proportion of children from households with low health literacy rose slightly to 21.2%. The prevalence of low parental health literacy was similar in the children lost to follow-up (21.2%) as in those retained (20.0%). However, the children lost to follow-up were older than the children included in the analysis, 10.4 and 9.8 years, respectively. The children lost to follow-up also had worse biomarker levels and came from households with slightly lower parental SES than the children included in our analysis.

**Table 1:** Description of the study population, stratified by those who were included in the study and those who were aged 5–15 years old during the baseline assessment (T1) but were lost to follow-up

		Children included in the analysis (*n* = 6683)		Eligible children lost to follow-up (*n* = 3086)		
		Mean (SD), median (IQR) or *n* (%)	*N*	Mean (SD), median (IQR), or *n* (%)	*N*	*P*-value^a^
Sex at T1	Male	3299 (49.4%)	6683	1505 (48.8%)	3086	0.586
	Female	3384 (50.6%)		1581 (51.2%)		
Age at T1 (years)		9.8 (2.6)	6683	10.4 (3.1)	3086	<0.001
Average parental age at T1 (years)		40.8 (4.6)	6683	40.9 (5.0)	3086	0.330
Baseline (T1) MetS components:						
Fasting glucose (mmol/l)		4.65 (0.43)	4271	4.69 (0.42)	1854	<0.001
High-density lipoprotein (mmol/l)		1.59 (0.34)	4440	1.56 (0.33)	1937	<0.001
Triglycerides (mmol/l)		0.61 (0.46–0.82)	4440	0.64 (0.49–0.86)	1937	<0.001
Mean arterial pressure (mmHg)		77.23 (6.66)	5299	78.29 (6.83)	2442	<0.001
Waist circumference (cm)		64.65 (8.16)	5310	67.20 (9.05)	2445	<0.001
Continuous MetS score at T4		–0.004 (2.781)	5048	–	–	–
Parental health literacy at T3 (maternal)	Low	1114 (20.0%)	5571	430 (21.6%)	1991	0.128
	Adequate	4457 (80.0%)		1561 (78.4%)		
Parental health literacy at T3 (average)	Low	1181 (21.2%)	5571	421 (21.1%)	1991	0.975
	Adequate	4390 (78.8%)		1570 (78.9%)		
Educational level at T1 (in years)		12 (12–15)	6649	12 (12–15)	3064	<0.001
Income at T1^b^		1360.53 (406.68)	6060	1329.46 (423.46)	2793	0.006
Occupational level at T1^c^		49.3 (12.1)	6635	48.3 (12.5)	3057	<0.001

a *P*-values from testing the difference between the two groups using Fisher’s exact test for categorical variables or Kruskal–Wallis test for numerical variables.

b Equivalized household income: calculated as the net household income in Euros divided by the square root of the number of individuals who live off of the income.

c Occupation: measured using the standard international occupational prestige scale, which is a continuous measure of occupation. It focuses on the prestige an occupation gives its holder, not on the incomes associated with occupations.

### SES and cMetS: relationships and mediation by parental health literacy

Higher levels of parental SES were related to lower cMetS scores, and this was partially mediated by parental health literacy (table 2). If parental education were 4 years longer, e.g. university instead of secondary school, cMetS scores were on average 0.499 [95% confidence interval (CI): 0.364–0.635] units lower, which is a small effect (*d*: 0.18). Similarly, if parental income and occupational level would be 1 SD higher, cMetS scores were on average 0.136 and 0.196 units lower, respectively. The effects of parental income and occupation on cMetS were small (*d*: 0.05 and 0.07, respectively). Parental health literacy mediated the relationship between the individual measures of SES (education, income and occupation) and MetS by 6.7%, 11.8% and 8.3%, respectively. The total effects remained the same in the sensitivity analyses. However, the percentages mediated by parental health literacy, which was based on the average of both parents health literacy, increased to 10.5% (education), 17.5% (income) and 12.5% (occupation).

**Table 2:** The estimated mean difference in cMetS effects of SES and parental health literacy on cMetS

	cMetS—primary analysis^e^ [mean difference (95%CI)]	cMetS—sensitivity analysis [mean difference (95%CI)]
Education^a^		
Direct effect	–0.466 (–0.602 to –0.330)	–0.447 (–0.584 to –0.310)
Indirect effect of parental health literacy	–0.033 (–0.065 to –0.001)	–0.052 (–0.092 to –0.013)
Total effect	–0.499 (–0.635 to –0.364)	–0.499 (–0.635 to –0.364)
Proportion mediated^b^	6.7%	10.5%
Income^c^		
Direct effect	–0.120 (–0.201 to –0.038)	–0.112 (–0.193 to –0.031)
Indirect effect of parental health literacy	–0.016 (–0.028 to –0.004)	–0.024 (–0.037 to –0.010)
Total effect	–0.136 (–0.219 to –0.052)	–0.136 (–0.219 to –0.052)
Proportion mediated^b^	11.8%	17.5%
Occupation^d^		
Direct effect	–0.180 (–0.265 to –0.095)	–0.171 (–0.257 to –0.086)
Indirect effect of parental health literacy	–0.016 (–0.029 to –0.004)	–0.025 (–0.040 to –0.009)
Total effect	–0.196 (–0.284 to –0.108)	–0.196 (–0.284 to –0.108)
Proportion mediated^b^	8.3%	12.5%

a Education: measured using the minimum number of years of education required to attain their highest level of education. The reference level is 12 years of education (secondary school), and the treatment level is 16 years of education (university education).

b Due to rounding, the proportion mediated may differ from the value calculated using the indirect and total effect presented in the table.

c Equivalized household income: calculated as the net household income in Euros divided by the square root of the number of individuals who live off the income. The reference level is the mean income level, and the treatment level is 1 SD above the mean.

d Occupation: measured using the standard international occupational prestige scale, which is a continuous measure of occupation. It focuses on the prestige an occupation gives its holder, not on the incomes associated with occupations. The reference level is the mean occupation level, and the treatment level is 1 SD above the mean.

e Parental health literacy was primarily measured using maternal data in the primary analysis, and it was measured using an average of both parent’s data in the sensitivity analysis.

## Discussion

We assessed to what extent parental health literacy mediates the relationships between different measures of parental SES and paediatric MetS. We found that higher parental SES resulted in slightly lower cMetS scores, and this was partially mediated by parental health literacy. The proportion mediated by parental health literacy varied across indicator of SES; it was the largest for income. Additionally, the percentage mediated was higher when parental health literacy was defined using the average of both parents’ health literacy, but the pattern of our findings remained the same.

The extent to which health literacy mediated the relationship between SES and MetS was limited; the proportion mediated was largest for income (11.8%). To the best of our knowledge, this is the first study to assess this mediating pathway. Importantly, we have done so in a robust manner by using a causal mediation analysis. Our findings are supported by a recent review which found that health literacy mediates socioeconomic inequalities in adults.^8^ Given that education has the largest impact on health literacy,^8^ it is not surprising that the indirect effect was largest for education. However, we also expected the proportion mediated by health literacy to be largest for education, instead of for income. Despite the strong relationship between education and parental health literacy, other factors appear to mediate this relationship. Health literacy may be an important attribute, especially amongst individuals with low income, as it could result in making better lifestyle choices given one’s resources. Finally, it should be noted that the pattern of our findings was consistent in the sensitivity analysis, which used average parental health literacy instead of primarily using maternal health literacy. The proportion mediated increased across all indicators of SES when using average parental health literacy, and this may be explained by the fact that if only one parent has low health literacy, the other may be able to compensate. In short, parental health literacy explains a small portion of socioeconomic differences in paediatric MetS.

We found that the effect of SES on MetS was rather limited, confirming findings of other studies that also used cMetS scores.^2^^,^^27^ In the first study, it is difficult to gauge the size of the effect as the authors do not provide a standard deviation for their cMetS score.^2^ However, they found that cMetS scores at baseline were on average 0.28 units higher for children whose parents had low educational levels than for children whose parents had high educational levels. The other study, on a sub-sample (*n* = 1217) of the children included in the present study, only found a small effect of parental education on cMetS.^27^ The larger sample used in the present study may have provided additional power to detect the relationship between both parental income and occupation and MetS compared with that study.

### Strengths and limitations

This study’s strengths lie in the quality of its data and the robustness of our findings. Due to the community-based nature, standardized protocols and longitudinal design of Lifelines, we were able to obtain high-quality data for a large sample of children that are generally representative of this region of the Netherlands.^11^^,^^12^ Using multiple indicators of SES to account for their unique relationships with health and the use of a causal mediation analysis added to the robustness of our findings. Furthermore, our sensitivity analysis, which had the same pattern of results as our primary analysis, demonstrated the robustness of our results.

This study also has some limitations. First, the children lost to follow-up had slightly worse biomarkers and came from households with slightly lower SES at baseline than the children included in our study. This loss to follow-up may have introduced some bias, but it is unlikely to have had a major impact given the differences between the two groups were rather small. Additionally, we may have underestimated the full effect of health literacy as our measure of health literacy focused primarily on functional health literacy, which is a specific component of health literacy. More comprehensive measures of health literacy that include other components of health literacy, e.g. critical health literacy and communicative health literacy, may have led to a stronger mediating effect. Nonetheless, we used a validated questionnaire for health literacy^13^ that has been used in other studies.^14^^,^^15^

### Implications

This study indicates that interventions which aim to improve parental health literacy may partially reduce inequalities in paediatric MetS. Health literacy can be targeted at various levels. For example, a recent study found that using a health literacy intervention to target both the children’s parents and their health care providers resulted in less weight gain during the first 18 months of life.^28^ Additionally, the children themselves can be taught about health literacy during their schooling, and this has the potential for many benefits throughout the life course.^29^ However, further research is needed into the potential impact of improving health literacy in children. Additionally, our results demonstrate that the effect of health literacy differs when defined using mostly maternal data vs. the average of both parents. Future studies should take this into consideration, and we also suggest studies should investigate the role of paternal health literacy. Finally, previous research has shown that health literacy impacts various aspects of children’s health,^9^ so it is likely that interventions targeting parental health literacy will influence various socioeconomic health inequalities.

## Conclusion

Socioeconomic inequalities in paediatric MetS are relatively small, being largest by parental education. Targeting parental health literacy may not have a major impact on socioeconomic inequalities in paediatric MetS. Additional research is needed into the mediating role of parental health literacy on other socioeconomic health inequalities in children.

## Data Availability

Researchers can apply to use the Lifelines data used in this study. More information about how to request Lifelines data and the conditions of use can be found on their website (https://www.lifelines.nl/researcher/how-to-apply). Key points Parental socioeconomic status (SES) has a small inverse relationship with paediatric metabolic syndrome (MetS) Parental health literacy partially mediates the relationship between parental SES and paediatric MetS. Further research is needed into the mediating role of parental health literacy with other outcomes.
